# Emotion Regulation Across Adulthood: New Research Directions

**DOI:** 10.1093/geroni/igaf122.635

**Published:** 2025-12-31

**Authors:** Claudia Haase, Joseph Mikels

## Abstract

Emotion regulation plays an important role in late-life well-being and health. While existing research has yielded important insights, numerous questions remain open. This symposium brings together four contributions to elucidate new directions in emotion regulation research across adulthood – featuring diverse emotion regulation and coping processes (e.g., acceptance, avoidance), contexts (e.g., daily stressors, repeated social stress exposure, caregiving), and study designs (e.g., laboratory-based, survey-based, daily diary, and longitudinal approaches). Dr. Haase and colleagues will present findings from a research program on acceptance (i.e., embracing emotions and thoughts without judgment). Laboratory-based and survey-based findings show that acceptance benefits physiological and mental health and becomes increasingly important in late life. Dr. Charles and colleagues will share insights into belonging from a large-scale national study of daily experiences in midlife and later adulthood. Findings show that belonging is associated with lower negative affect and buffers the effects of daily stressors on negative affect. Dr. Luong will present insights from a laboratory-based study with younger and older adults who were repeatedly exposed to social stress. While older adults use effective emotion regulation strategies (i.e., cognitive reappraisal) more frequently, they also show greater affective reactivity to repeated social stress exposure. Gad and colleagues will share findings from a longitudinal study with adult-child caregivers of parents with memory loss. Findings show that avoidant coping predicts greater caregiver burden concurrently and longitudinally, but caregivers who experience more positive emotions relative to their negative emotions may be buffered. The symposium will conclude with a discussion from Dr. Mikels.

